# Neutral effects of SGLT2 inhibitors in acute coronary syndromes, peripheral arterial occlusive disease, or ischemic stroke: a meta-analysis of randomized controlled trials

**DOI:** 10.1186/s12933-023-01789-5

**Published:** 2023-03-13

**Authors:** Pei-Chien Tsai, Wei-Jung Chuang, Albert Min-Shan Ko, Jui-Shuan Chen, Cheng-Hsun Chiu, Chun-Han Chen, Yung-Hsin Yeh

**Affiliations:** 1 Department and Graduate Institute of Biomedical Sciences, Chang Gung University, No. 259, Wenhua 1st Rd, Guishan Dist., Taoyuan City, 33302 Taiwan; 2 Master’s Program in Clinical Trials and Assessment, Department of Biomedical Sciences, Chang Gung University, No. 259, Wenhua 1st Rd, Guishan Dist., Taoyuan City, 33302 Taiwan; 3 Molecular Infectious Disease Research Center, Chang Gung Memorial Hospital, No. 5, Fuxing st., Guishan Dist., Taoyuan City, 333 Taiwan; 4 Healthy Aging Research Center, Chang Gung University, No. 259, Wenhua 1st Rd, Guishan Dist., Taoyuan City, 33302 Taiwan; 5 Cardiovascular Department, Chang Gung Memorial Hospital, No. 5, Fuxing st., Guishan Dist., Taoyuan City, 333 Taiwan; 6 Division of Pediatric Infectious Diseases, Department of Pediatrics, Chang Gung Memorial Hospital, No. 5, Fuxing st., Guishan Dist., Taoyuan City, 333 Taiwan; 7 School of Medicine, Chang Gung University, No. 259, Wenhua 1st Rd, Guishan Dist., Taoyuan City, 33302 Taiwan

**Keywords:** SGLT2 inhibitor, Canagliflozin, Dapagliflozin, Empagliflozin, Ertugliflozin, Meta-analysis, Type 2 diabetes, Acute coronary syndrome, Peripheral arterial occlusive disease, Ischemic stroke

## Abstract

**Background:**

Patients with type 2 diabetes are at increased risk for cardiovascular diseases. Sodium-glucose transport 2 inhibitors (SGLT2i) have been shown to enhance cardiovascular health since their debut as a second-line therapy for diabetes. Acute coronary syndrome (ACS), peripheral arterial occlusive disease (PAOD), and ischemic stroke (IS) are types of atherosclerotic cardiovascular disease (ASCVD), although the benefits of treating these disorders have not been shown consistently.

**Methods:**

We searched four databases (PubMed, Embase, the Cochrane library, and clinicaltrial.gov) for randomized clinical trials (RCTs) until November of 2022. Comparisons were made between SGLT2i-treated and control individuals with type 2 diabetes. Primary outcomes were ACS, PAOD, and IS; secondary outcomes included cardiovascular mortality and all-cause mortality. Risk ratio (RR) and 95% confidence intervals (CI) were determined using a fixed effects model. Cochrane's risk-of-bias (RoB2) instrument was used to assess the validity of each study that met the inclusion criteria.

**Results:**

We enrolled 79,504 patients with type 2 diabetes from 43 RCTs. There was no difference in the risk of ACS (RR = 0.97, 95% CI 0.89–1.05), PAOD (RR = 0.98, 95% CI 0.78–1.24), or IS (RR = 0.95, 95% CI 0.79–1.14) among patients who took an SGLT2i compared to those who took a placebo or oral hypoglycemic drugs. Subgroup analysis revealed that none of the SGLT2i treatments (canagliflozin, dapagliflozin, empagliflozin, and ertugliflozin) significantly altered outcomes when analyzed separately. Consistent with prior findings, SGLT2i reduced the risk of cardiovascular mortality (RR = 0.85, 95% CI 0.77–0.93) and all-cause mortality (RR = 0.88, 95% CI 0.82–0.94).

**Conclusion:**

Our results appear to contradict the mainstream concepts regarding the cardiovascular effects of SGLT2i since we found no significant therapeutic benefits in SGLT2i to reduce the incidence of ACS, PAOD, or IS when compared to placebo or oral hypoglycemic drugs.

**Supplementary Information:**

The online version contains supplementary material available at 10.1186/s12933-023-01789-5.

## Introduction

Treatment for type 2 diabetes should begin with metformin and other lifestyle adjustments, as recommended by the American Diabetes Association [1]. Sodium-glucose transport 2 inhibitors (SGLT2i) and other second-line therapeutic agent combinations may be necessary if first-line treatment fails to bring blood glucose under control. SGLT2 is a sodium-glucose transporter that is found in the S1 segment of the proximal tubule. SGLT2i aids in maintaining healthy blood glucose levels by blocking SGLT2 reabsorption [2]. The four most widely used SGLT2i, canagliflozin, dapagliflozin, empagliflozin, and ertugliflozin, all bind to the SGLT2 protein with varying degrees of affinity [3].

Stable control of blood glucose is just one of the benefits of SGLT2i. It has been reported that among adults with diabetic kidney disease, SGLT2i are associated with reduced risks of major adverse cardiovascular events (MACE), kidney outcomes, hospitalization for heart failure, and death [4, 5]. In addition, SGLT2i decreases systolic blood pressure in patients with heart failure [6], yielding also benefits in patients with heart failure with preserved ejection fraction [7]. SGLT2i act as anti-inflammatory agents by either indirectly improving metabolism and reducing stress conditions or via direct modulation of inflammatory signaling pathways [8]; the direct cardiac effects seem to be mediated by modulation of intracellular sodium concentration via the sodium-interactome [9].

Up to two thirds of patients with type 2 diabetes have atherosclerotic cardiovascular disease (ASCVD) [10], making them less manageable and leading to worse outcomes than the general population [11]. Animal models propose that SGLT2i prevents ASCVD by lowering serum levels of inflammatory factors linked to atherosclerosis, stopping the proliferation and migration of vascular smooth muscle cells (VSMCs), blocking foam cell formation, preventing platelet activation, and improving autophagy impairment [12], but human clinical data is less conclusive. To further understand the relationship between SGLT2i cardiovascular impact and ASCVD events, especially ACS, PAOD, IS, and mortality outcomes in individuals with type 2 diabetes, we conducted a meta-analysis.

## Methods

### Database sources and search strategy

**Fig. 1:**
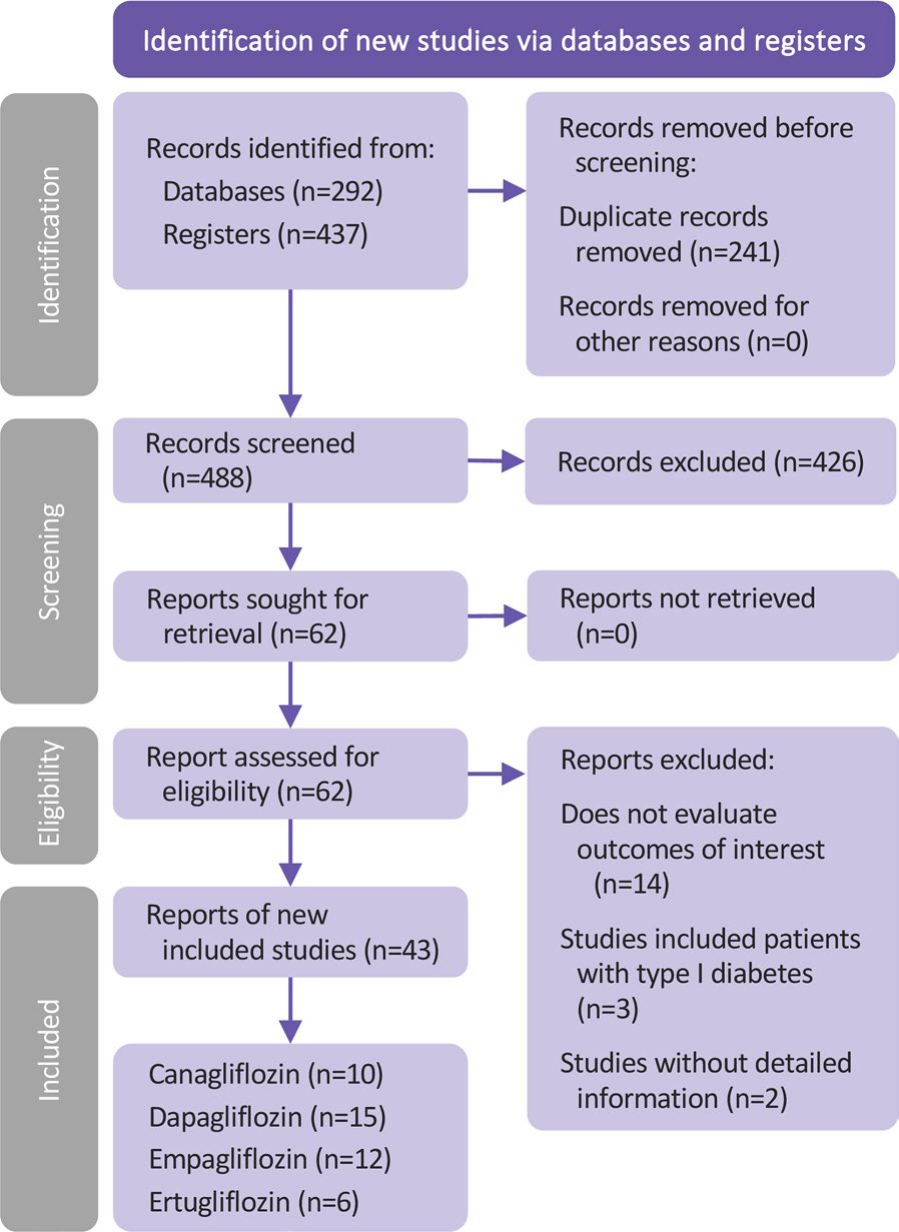
Study workflow of finding and including literature

This study followed the PRISMA (Preferred Reporting Items for Systematic Reviews and Meta-analysis) guidelines [13] and the Cochrane Handbook (Version 6.1) [14] in terms of its methodology, including data sources, inclusion and exclusion criteria, outcome assessment, quality assessment, and use of statistical methods. Four international databases (PubMed, Embase, Cochrane Library, and ClinicalTrials.gov) were searched. Terminology used to described “type 2 diabetes”, “sodium-glucose cotransporter-2 inhibitors,” and terms relevant to “acute coronary syndrome,” “peripheral arterial occlusive disease,” “ischemic stroke,” “cardiovascular mortality” and “all-cause mortality” were searched in the databases. The database search algorithm is provided in Additional file 1. The data collection workflow is shown in Fig. 1. The last search time was conducted in November 2022. In the first phase of the literature search, we retrieved a total of 729 records (292 from databases and 437 from registries), after removing 241 duplicates, we screened 488 records; then, 426 records were excluded based on the exclusion criteria for this study; finally, we retrieved 62 records and aseesed their eligibility, and we ended up including 43 studies.

### Inclusion and exclusion criteria

Our inclusion criteria were studies reported in English, have a comprehensive documentation of their outcomes, and patients with type 2 diabetes who were 18 or older. The exclusion criteria were studies involving patients with type 1 diabetes or malignancies, letters to the editor, editorials, case reports, review articles, and literature based on animal model. As glucagon-like peptide-1 (GLP-1) is an effective treatment for managing blood glucose levels, we also ruled out trials in which GLP-1 drugs were used as a control. Our control group was defined as those receiving placebo or active therapy using oral hypoglycemic drugs. In order to analyze the occurrence of adverse events in a larger pool of patients with type 2 diabetes, the placebo- and active-controlled trials were merged.

Two independent reviewers (WJC and RXC) were involved in the literature search and citation eligibility review, while a third reviewer (CHC) crossed-checked all eligible references. Final eligibility of references was determined by two senior authors (PCT and YYH). YYH carefully reviewed the definition of results and the use of SGLT2i in each study.

### Outcome measures

The primary outcome was the incidence of ACS (defined as acute myocardial infarction and/or unstable angina), PAOD (not including other related events such as peripheral artery ischemia or peripheral artery embolism), and IS. Secondary outcomes were cardiovascular mortality and all-cause mortality. The incidence of adverse events was retrieved from the clinicaltrials.gov registry and the published studies.

### Data extraction and quality assessment

Primary and secondary outcomes, study characteristics (sample size, trial name, ClinicalTrials.gov identifier), treatment details (dose, follow-up duration, protocol), patient characteristics (age, sex) were extracted from all included studies. Different doses of the same drug were pooled into one treatment group. To avoid duplication from the same population, only the most recent randomized controlled trials (RCTs) with the largest sample size were considered. Methodological quality was assessed using version 2 of the Cochrane risk-of-bias tool for randomized trials (RoB2) [15]. Selective reporting, random sequence generation, other sources of bias, incomplete outcome data, blinded outcome assessment, and allocation concealment were all identified as potential sources of bias. Each risk of bias category was rated as low, high, and unclear. The RoB 2 was used to evaluate the reliability of the evidence. Study selection, data extraction, and quality assessment of data extraction were carried out by three independent reviewers (WJC, RXC, and CHC). The data gathered from the publications was analyzed for potential bias by two additional researchers (PCT and YYH), who discussed their contrasting findings until a consensus is reached.

### Statistical analysis

Funnel plots and Egger’s test [16] were used to look for signs of publication bias. Pooled risk ratio (RR) and 95% confidence intervals (CI) were used to analyze the incidence of ACS, PAOD, and IS patients receiving conventional therapy with SGLT2i or oral hypoglycemic drugs. The Higgins and Thompson *I*^*2*^ statistic and the Cochrane Q test were used to analyze the degree of study heterogeneity. The level of statistical significance for the Q test was set at a P-value < 0.1. In principle, if *I*^*2*^ was greater than 50%, a random-effects model was used for meta-analysis, otherwise, a fixed effects model was used. In order to better account for any clinical background heterogeneity, we also adopted a random effects model for all analyses in the study. We ran subgroup analyses to look at the impact of individual types of SGLT2i treatment, because different SGLT2i treatments might produce different outcomes. In all analyses, P-values < 0.05 (two-sided) were considered statistically significant. All analyses and visualizations were generated with R version 4.1.2 and R packages *meta* and *dmetar*. Trial sequential analysis (TSA) was used to estimate the required sample size to reach 80% study power based on the incidence rate in control group and the relative risk reduction rate obtained from each meta-analysis [17]. The parameters of the study were calculated to be an alpha level of 0.05 and a power of 80%. TSA was performed using TSA 0.9.5.10 Beta.

## Results

Figure 1 shows the final 43 RCTs with a total of 79,504 patients with type 2 diabetes; 48,568 patients received SGLT2i in combination with background treatment, whereas 30,936 patients used placebo or oral hypoglycemic drugs. Four of the 43 studies have not yet been published. In terms of the types of SGLT2i treatments, these were canagliflozin (10 studies), dapagliflozin (15 studies), empagliflozin (15 studies), and ertugliflozin (6 studies).

### Baseline characteristics of included studies

Table 1 shows the baseline characteristics of the included studies. All eligible RCTs were published between 2010 and 2020. Median follow-up time was 1.9 years, sample size ranged from 218 to 17,143 participants, and 21.4% to 54.5% were female. First-line medications most often used to treat diabetes were metformin (58.1%), sulfonylurea (20.9%), and insulin (18.6%). The risk of bias in the 43 studies is shown in Additional file 2. Incomplete data on ClinicalTrials.gov means that there may be minor problems with certain studies, and around half of the studies had a low risk of bias (see Additional file 3). Five of the 43 studies contain evidence of atherosclerotic cardiovascular disease (ASCVD) at baseline, such as associated high cardiovascular risk [18–20], cerebrovascular disease or high blood pressure [21], and atherosclerosis in the coronary, cerebral, or peripheral vascular systems [22]. Subgroup analyses were used to examine the effect of ASCVD history or evidence on the overall results. Subgroup analyses show that the incidence rates of ACS, PAOD, and IS are similar across the two subgroups (5 vs. 38 studies), and that the risk ratios are consistent with the overall results of this study (see Additional file 4).

**Table 1:** Characteristics of the included randomized clinical trials in this study

Study	Number of patients (M/F)		Mean age (SD)		Interventions	Background therapy
	Treatment	Control	Treatment	Control		
Lavalle-González et al., 2013 [33]	1101 (705/396)	549 (266/283)	55.4 (9.3)	54.7 (9.7)	Canagliflozin (100/300 mg)/Placebo and Sitagliptin	Metformin
Cefalu et al., 2013 [34]	968 (493/475)	482 (238/244)	58.9 (9.4)	56.3 (9.0)	Canagliflozin (100/300 mg)/Glimepiride	Metformin
NCT01106690, 2013	227 (140/87)	115 (76/39)	56.9 (10.3)	58.3 (9.6)	Canagliflozin (100/300 mg)/Placebo and Sitagliptin	Metformin and pioglitazone
Yale et al., 2014 [35]	179 (106/73)	90 (57/33)	68.7 (8.2)	68.2 (8.4)	Canagliflozin (100/300 mg)/Placebo	Accordance with local guidelines
Neal et al., 2015 [20]	2886 (1903/983)	1441 (955/486)	62.2 (8.1)	62.3 (7.9)	Canagliflozin (100/300 mg)/Placebo	Sulfonylurea
Bode et al., 2015 [36]	477 (253/224)	237 (143/94)	64.3 (6.3)	63.2 (6.2)	Canagliflozin (100/300 mg)/Placebo	Stable antihyperglycemic (AHA) regimen
Rosenstock et al., 2016 [37]	949 (453/496)	237 (116/121)	54.9 (9.9)	55.2 (9.8)	Canagliflozin (100/300 mg)/Metformin	Metformin
NCT01989754, 2017	2904 (1851/1053)	2903 (1792/1111)	63.9 (8.4)	64 (8.3)	Canagliflozin (100 mg 13 weeks then 300 mg)/Placebo	Accordance with local guidelines
Perkovic et al., 2019 [23]	2200 (1438/762)	2197 (1465/732)	62.9 (9.2)	63.2 (9.2)	Canagliflozin (100 mg)/ Placebo	Accordance with local guidelines
Lingvay et al., 2019 [38]	394 (201/193)	392 (221/171)	57.5 (10.7)	55.7 (11.1)	Canagliflozin (100 mg 13 weeks then 300 mg)/Semaglutide	Metformin
Nauck et al., 2011 [39]	406 (227/179)	408 (227/181)	58.1 (9.4)	58.6 (9.8)	Dapagliflozin (not mentioned)/Glipizide	Metformin
Strojek et al., 2011 [40]	450 (217/233)	146 (72/74)	59.7 (9.4)	60.3 (10.2)	Dapagliflozin (2.5/5/12 mg)/Placebo	Glimepiride
Henry et al., 2012 [41]	827 (657/170)	409 (314/95)	51.5 (10.3)	52.3 (10.1)	Dapagliflozin (5/10 mg)/Metformin	Metformin
Bailey et al., 2013 [42]	409 (216/193)	137 (76/61)	54 (NA)	53.7 (NA)	Dapagliflozin (2.5/5/10 mg)/Placebo	Metformin
Leiter et al., 2014 [43]	482 (323/159)	483 (324/159)	63.9 (7.6)	63.6 (7.0)	Dapagliflozin (10 mg)/Placebo	Usual care
NCT01137474, 2014	633 (358/275)	311 (171/140)	NA (NA)	NA (NA)	Dapagliflozin (2.5/5/10 mg)/Placebo	OAD with or without insulin
Wilding et al., 2014 [44]	610 (290/320)	197 (99/98)	59.8 (8.1)	58.8 (8.6)	Dapagliflozin (2.5/5/10 mg)/Placebo	Insulin
Cefalu et al., 2015 [21]	460 (314/146)	462 (318/144)	62.8 (7.0)	63 (7.7)	Dapagliflozin (10 mg)/Placebo	Stable background treatment except rosiglitazone
Bailey et al., 2015 [45]	410 (198/212)	75 (31/44)	NA (NA)	52.7 (10.3)	Dapagliflozin (2.5/5/11 mg)/Placebo	Metformin
Matthaei et al., 2015 [46]	109 (47/62)	109 (61/48)	61.1 (9.7)	60.9 (9.2)	Dapagliflozin (10 mg)/Placebo	Metformin and sulfonylurea
Müller-Wieland et al., 2018 [47]	313 (201/112)	312 (207/105)	57.4 (9.4)	58.6 (8.4)	Dapagliflozin (13 mg)/Glimepiride	Metformin
Scott et al., 2018 [48]	306 (186/120)	307 (169/138)	66.6 (8.6)	67.7 (8.5)	Dapagliflozin (5 mg titrated to 10 mg)/Sitagliptin plus Placebo Dapagliflozin	Metformin with or without sulfonylurea
Fioretto et al., 2018 [49]	160 (91/69)	161 (91/70)	65.3 (6.2)	66.2 (6.5)	Dapagliflozin (12 mg)/Placebo	Insulin, metformin, sulfonylurea or TZD
Yang et al., 2018 [50]	139 (66/73)	133 (64/69)	56.5 (8.4)	58.6 (8.9)	Dapagliflozin (10 mg)/Placebo	Insulin
Wiviott et al., 2019 [19]	8574 (5403/3171)	8569 (5319/3250)	63.9 (6.8)	64 (6.8)	Dapagliflozin (10 mg)/Placebo	Current background therapy
Häring et al., 2013 [51]	1042 (568/474)	431 (227/204)	55.4 (9.9)	56 (9.7)	Empagliflozin (10/25 mg)/Placebo	Metformin or sulfonylurea
Ferrannini et al., 2013 [52]	547 (277/270)	112 (57/55)	58.9 (8.6)	57.6 (9.8)	Empagliflozin (10 mg)/Sitagliptin and Metformin	Metformin
Barnett et al., 2014 [53]	419 (249/170)	319 (181/138)	63.7 (8.9)	64.1 (8.7)	Empagliflozin (10/25 mg)/Placebo	Metformin, insulin or sulfonylurea
Rosenstock et al., 2014 [54]	375 (181/194)	188 (75/113)	57.4 (9.1)	55.3 (10.1)	Empagliflozin (10/25 mg)/Placebo Empagliflozin 10 mg plus Placebo Empagliflozin 25 mg	Insulin or metformin
Zinman et al., 2015 [18]	4687 (3336/1351)	2333 (1680/653)	63 (8.6)	63.2 (8.8)	Empagliflozin (10/25 mg)/Placebo	Current background therapy
Roden et al., 2015 [55]	1325 (765/560)	877 (486/391)	56 (10.3)	55.7 (10.0)	Empagliflozin (10/25 mg)/Placebo and Sitagliptin	Metformin, sulfonylureas
NCT01649297, 2015	876 (483/393)	107 (55/52)	57.6 (10.2)	57.9 (11.2)	Empagliflozin (5/12.5 mg BID, 10/25 mg QD)/ Placebo	Metformin
Rosenstock et al., 2015 [56]	324 (186/138)	170 (90/80)	59.2 (10.2)	58.1 (9.4)	Empagliflozin (10/25 mg)/Placebo	Insulin
Araki et al., 2015 [57]	273 (195/78)	63 (47/16)	61.6 (9.8)	60 (10.2)	Empagliflozin (10/25 mg)/Metformin and Sulfonylurea	Sulfonylurea, biguanide, TZD, AGI or DPP-4
Hadjadj et al., 2016 [58]	1019 (593/426)	341 (187/154)	52.6 (11.0)	52.5 (10.9)	Empagliflozin (5/12.5 mg BID, 10/25 mg QD)/ Metformin	Metformin
Ridderstråle et al., 2018 [59]	765 (432/333)	780 (421/359)	56.2 (10.3)	55.7 (10.4)	Empagliflozin (25 mg)/Glimepiride plus Placebo Empagliflozin	Metformin
Rodbard et al., 2019 [60]	410 (209/201)	411 (206/205)	58 (10.0)	57 (10.0)	Empagliflozin (25 mg)/Semaglutide	Accordance with local guidelines
Pratley et al., 2018 [61]	498 (261/237)	247 (154/93)	55.1 (9.8)	54.8 (10.7)	Ertugliflozin (5/15 mg)/Sitagliptin	Metformin
Rosenstock et al., 2018 [62]	412 (190/222)	209 (98/111)	56.7 (8.8)	56.5 (8.7)	Ertugliflozin (5/15 mg)/Placebo plus Glimepiride	Glimepiride or insulin
Grunberger et al., 2018 [63]	313 (159/154)	154 (72/82)	67.1 (8.4)	67.5 (8.9)	Ertugliflozin (5/15 mg)/Placebo	Exception of metformin, rosiglitazone, and other SGLT2 inhibitors
Dagogo-Jack et al., 2018 [64]	309 (163/146)	153 (100/53)	59.4 (9.0)	58.3 (9.2)	Ertugliflozin (5/15 mg)/Placebo	Metformin or sitagliptin
Hollander et al., 2019 [65]	880 (409/471)	435 (222/213)	58.4 (9.8)	57.8 (9.2)	Ertugliflozin (5/15 mg)/Glimepiride	Metformin
Cannon et al., 2020 [22]	5493 (3860/1633)	2745 (1901/844)	64.4 (8.1)	64.4 (8.0)	Ertugliflozin (5/15 mg)/Placebo	Insulin, metformin and sulfonylurea

### Effect of SGLT2i on acute coronary syndrome

**Fig. 2:**
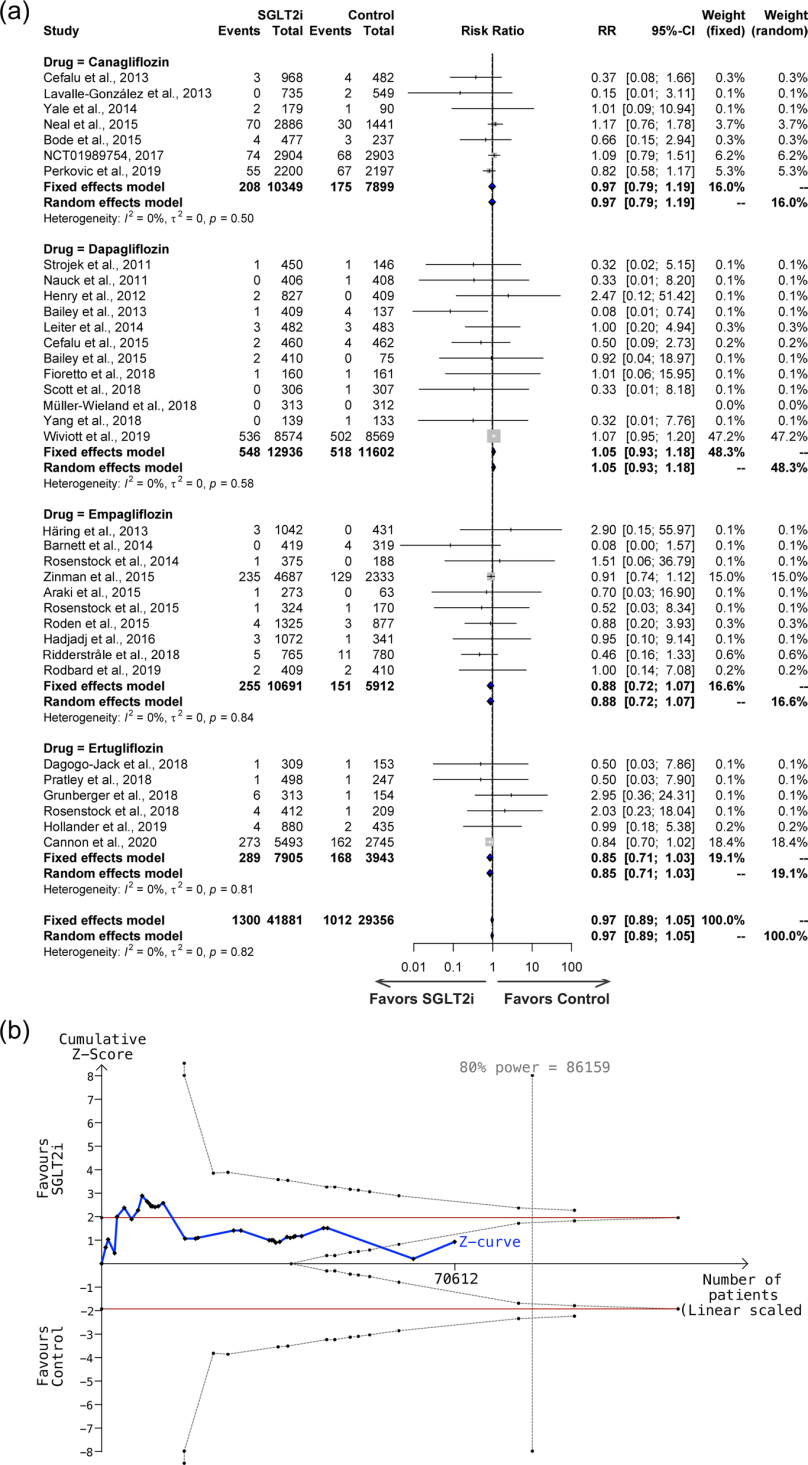
**a** Forest plot, and **b** Trial Sequential Analysis of effects of SGLT2i on acute coronary syndrome

A forest plot comparing the SGLT2i treatment for ACS to that of control is shown in Fig. 2a. Thirty-five studies, including canagliflozin (7 studies), dapagliflozin (12 studies), empagliflozin (10 studies), and ertugliflozin (6 studies) reported ACS as an adverse event, with 41,881 individuals in the SGLT2i group and 29,356 individuals in the control group, and an incidence of 3.10% in the SGLT2i group compared to 3.45% in the control group. There was no significant heterogeneity across the studies (*I*^2^ = 0%, P = 0.82 for the Q test) (Fig. 2a), and the overall risk ratio was not significant (RR = 0.97, 95% CI: 0.89–1.05). ACS did not differ significantly between the four SGLT2i medication groups and the control group: canagliflozin (RR = 0.97, 95% CI 0.79–1.19), dapagliflozin (RR = 1.05, 95% CI 0.93–1.18), empagliflozin (RR = 0.88, 95% CI 0.72–1.07), and ertugliflozin (RR = 0.85, 95% CI 0.71–1.03). A TSA was conducted using 34 studies with a total of 70,612 patients, a control group incidence rate of 3.45% and a relative risk reduction of 9.88% (Fig. 2b). Sequential analysis of trials indicates a sample size of 86,159 is needed to achieve 80% power, and there are not enough samples and effects for the cumulative Z-curve to pass the trial sequential monitoring boundaries.

### Effect of SGLT2i on peripheral arterial occlusive disease

**Fig. 3:**
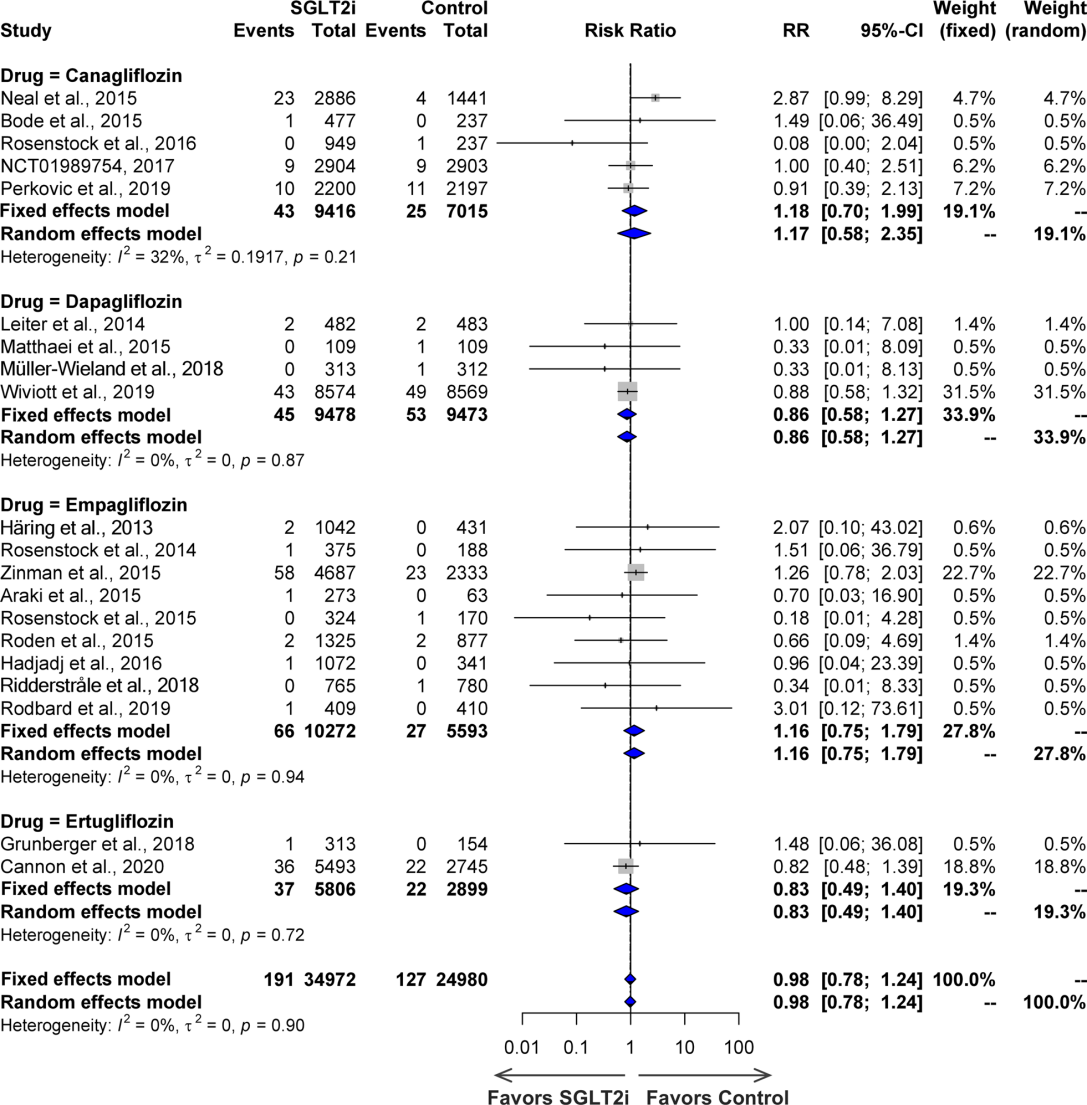
Forest plot of effects of SGLT2i on peripheral arterial occlusive disease

A forest plot depicting the SGLT2i treatment for PAOD to that of control is shown in Fig. 3. Twenty studies, including canagliflozin (5 studies), dapagliflozin (4 studies), empagliflozin (9 studies), and ertugliflozin (2 studies) reported PAOD as an adverse event, with a total of 34,972 individuals in the SGLT2i group and 24,980 individuals in the control group, and an incidence of 0.55% in the SGLT2i group compared to 0.51% in the control group. There was no significant heterogeneity across the studies (*I*^2^ = 0%, P = 0.90 for the Q test) (Fig. 3) and the risk ratio was not significant (RR = 0.98, 95% CI 0.78–1.24). Subgroup analysis revealed that PAOD did not differ between the four SGLT2i medication groups: canagliflozin (RR = 1.18, 95% CI: 0.70–1.99), dapagliflozin (RR = 0.86, 95% CI 0.58–1.27), empagliflozin (RR = 1.16, 95% CI 0.75–1.79), and ertugliflozin (RR = 0.83, 95% CI 0.49–1.40). A TSA was conducted using 20 studies with a total of 59,952 patients, a control group incidence of 0.51%, with a 7.27% decrease in relative risk for those who took preventative measures. To make any conclusions from the sequential analysis of trials, the sample size must be far larger than 59,952 in order to detect the relative risk reduction rate of 7.27% for peripheral arterial occlusive disease in the SGLT2i group compared with the control group with 80% power. Analysis shows that there are not enough samples and effects for the cumulative Z-curve to approach the trial sequential monitoring boundaries if the setting is set at 80% power.

### Effect of SGLT2i on ischemic stroke

**Fig. 4:**
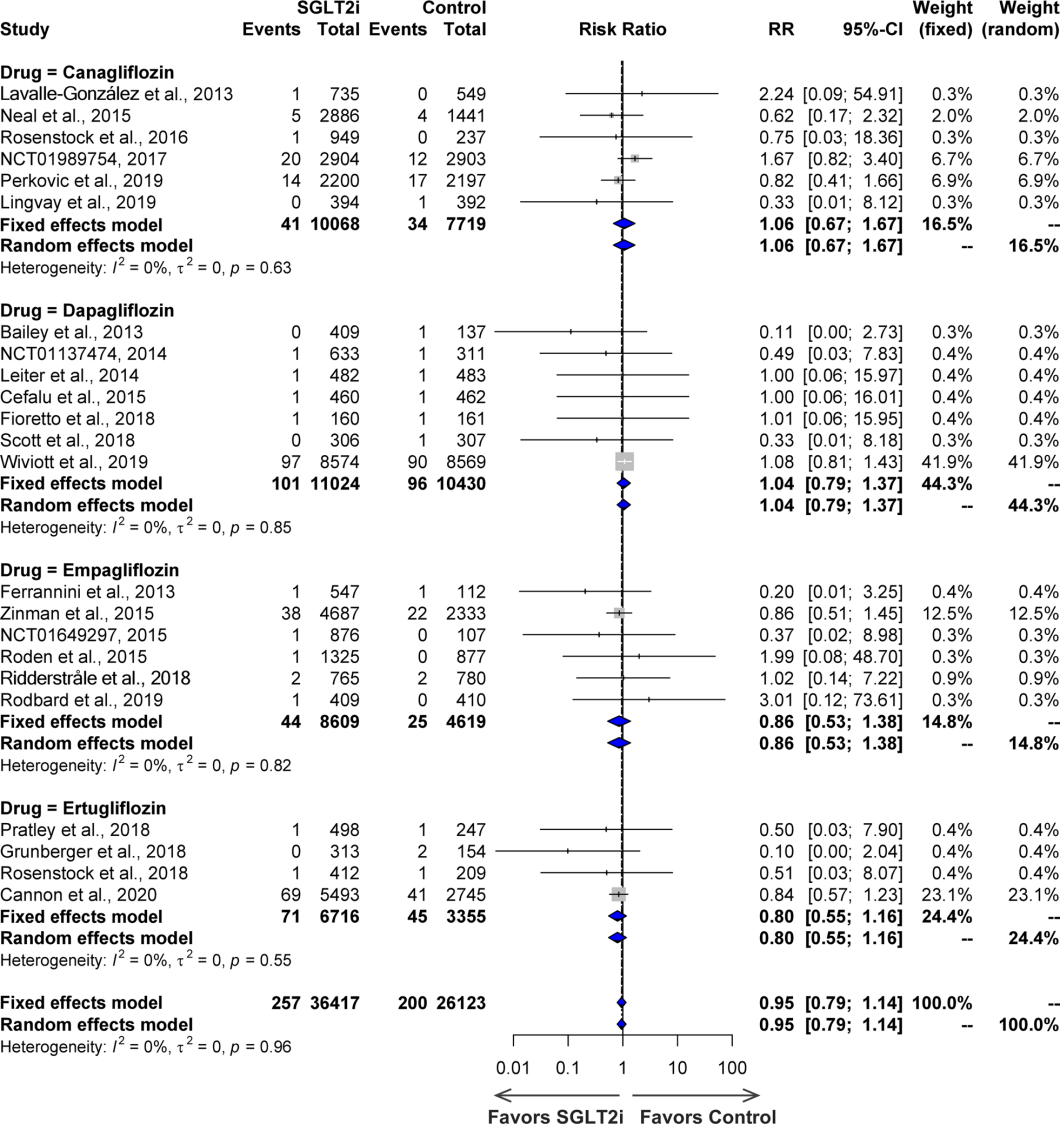
Forest plot of effects of SGLT2i on ischemic stroke

A forest plot is used to show how often SGLT2i causes IS compared to the controls (Fig. 4). IS was identified as an adverse event in 23 trials, including canagliflozin (6 studies), dapagliflozin (7 studies), empagliflozin (6 studies), and ertugliflozin (4 studies), with 36,417 individuals in the SGLT2i group and 26,123 individuals in the control group, and an incidence of 0.71% in the SGLT2i group compared to 0.77% in the control group. There was no significant heterogeneity across the studies (*I*^2^ = 0%, P = 0.96 for the Q test) (Fig. 4a). The overall risk ratio was not significant (RR = 0.95, 95% CI 0.79–1.14). Subgroup analysis revealed that IS did not differ significantly between the four SGLT2i medication groups and the control group: canagliflozin (RR = 1.06, 95% CI 0.67–1.67), dapagliflozin (RR = 1.04, 95% CI 0.79–1.37), empagliflozin (RR = 0.86, 95% CI 0.53–1.38), and ertugliflozin (RR = 0.80, 95% CI 0.55–1.16). A TSA was conducted using 23 studies with a total of 62,540 patients, a control group incidence rate of 0.77% and a relative risk reduction of 7.79%. To detect a 7.79% decrease in the risk of IS in the SGLT2i group compared with the control group and achieve 80% power, a sample size of more than 62,540 is needed. Analysis shows that there are not enough samples and effects for the cumulative Z-curve to approach the trial sequential monitoring boundaries if the setting is set at 80% power.

### Effect of SGLT2i on cardiovascular mortality and all-cause mortality

**Fig. 5:**
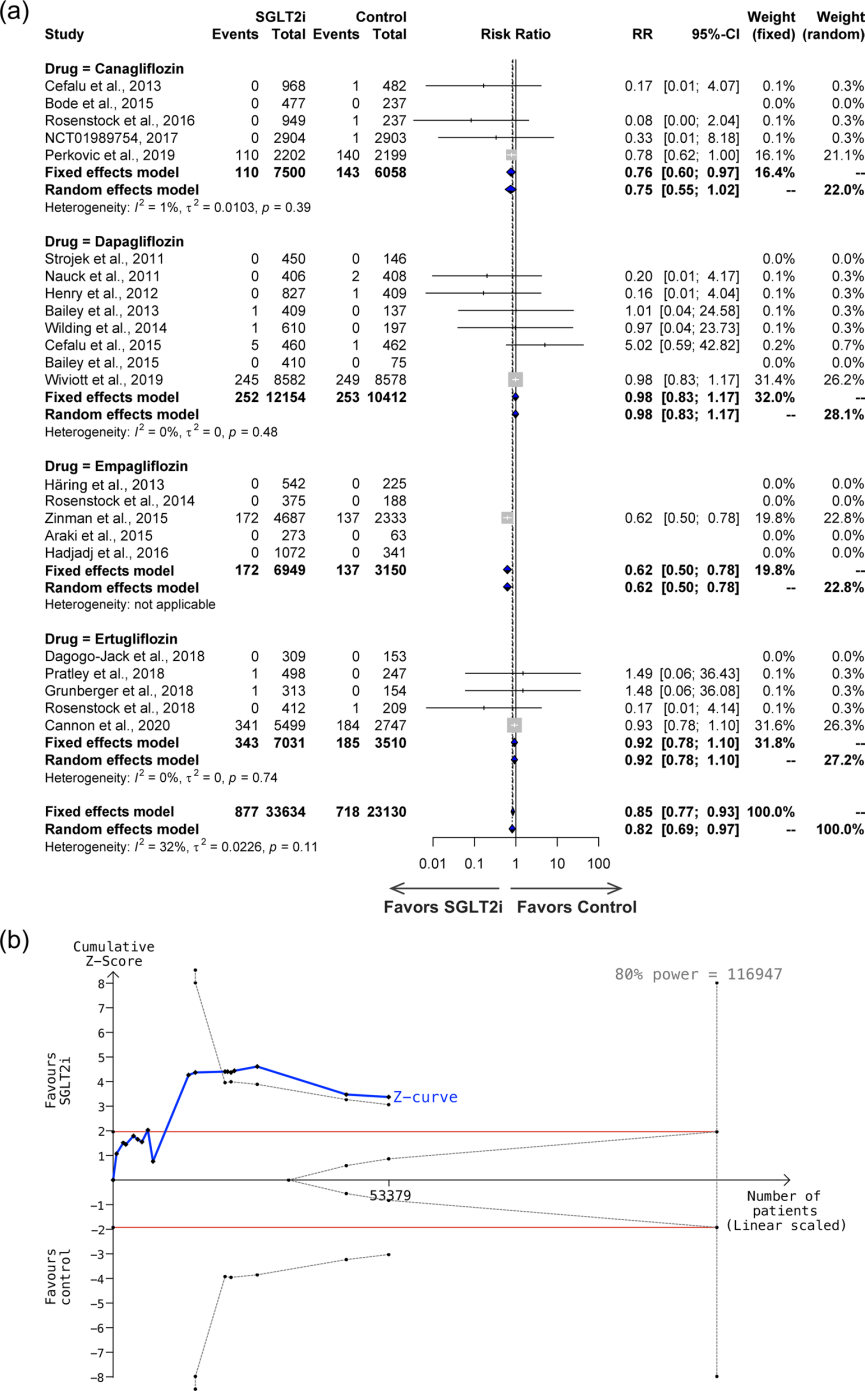
**a** Forest plot, and **b** Trial Sequential Analysis of effects of SGLT2i on cardiovascular mortality in 23 studies

Cardiovascular mortality was reported as an adverse event in 23 studies, including canagliflozin (5 studies), dapagliflozin (8 studies), empagliflozin (5 studies), and ertugliflozin (5 study), with a total of 33,634 individuals in the SGLT2i group and 23,130 individuals in the control group, and an incidence of 2.61% in the SGLT2i group compared to 3.10% in the control group. The included studies exhibited no heterogeneity (*I*^*2*^ = 32%, P = 0.11 for the Q test) (Fig. 5a). The overall risk ratio was significant (RR = 0.85, 95% CI 0.77–0.93). In the subgroup analysis, with the exception of canagliflozin (RR = 0.76, 95% CI 0.60–0.97) and empagliflozin that had lower risk ratios (RR = 0.62, 95% CI 0.50–0.78), dapagliflozin (RR = 0.98, 95% CI 0.83–1.17) and ertugliflozin (RR = 0.92, 95% CI 0.78–1.10) does not show a benefit for cardiovascular mortality (Fig. 5a). A TSA was conducted using 17 studies, a control group incidence rate of 3.1% and a relative risk reduction of 15.81% (Fig. 5b). Sequential analysis of trials indicates a sample size of 116,947 is required to reach 80% power. Although the included samples size was 53,379, the cumulative Z-curve already surpassed trial sequential monitoring boundaries, providing statistical power for the considerable protective impact of SGLT2i on cardiovascular mortality.

**Fig. 6:**
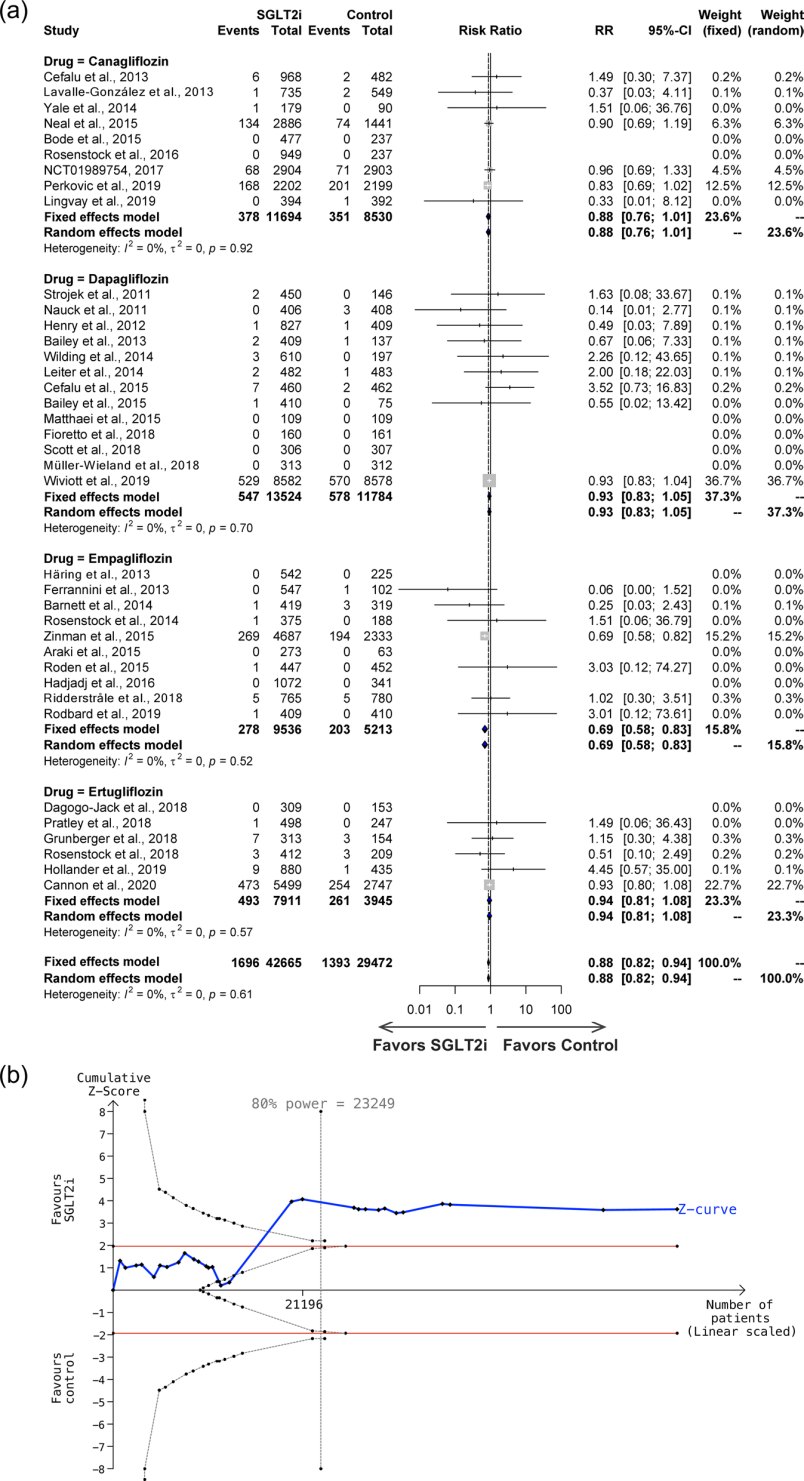
**a** Forest plot, and **b** Trial Sequential Analysis of effects of SGLT2i on all-cause mortality in 38 studies

There was a total of 38 studies, including canagliflozin (9 studies), dapagliflozin (13 studies), empagliflozin (10 studies), and ertugliflozin (6 studies) that looked at all-cause mortality in adverse events, with a total of 42,665 individuals in the SGLT2i group and 29,472 individuals in the control group, and an incidence of 3.98% in the SGLT2i group compared to 4.73% in the control group. There was no significant heterogeneity across the studies (*I*^2^ = 0%, P = 0.61 for the Q test) (Fig. 6a). The overall risk ratio was significant (RR = 0.88; 95% CI 0.82–0.94). Subgroup analysis also showed that, with the exception empagliflozin (RR = 0.69; 95% CI: 0.58–0.83) had lower risk of all-cause mortality, canagliflozin (RR = 0.88; 95% CI 0.76–1.01), dapagliflozin (RR = 0.93; 95% CI 0.83–1.05), and ertugliflozin (RR = 0.94; 95% CI 0.81–1.08) showed similar effect to control. A TSA was conducted using 30 studies with a total of 63,108 patients, a control group incidence rate of 4.73% and a relative risk reduction of 15.86% (Fig. 6b). Sequential analysis of trials indicates a sample size of 23,249 is required to reach 80% power, which is satisfied by the included studies, thus the cumulative Z-curve reached the trial sequential monitoring boundaries, demonstrating the significant protective effect of SGLT2i on all-cause mortality.

### Publication bias

Egger tests showed no publication bias, and the distribution of publications on the funnel plots for each meta-analysis was symmetrical (Additional file 5), suggesting that publication bias in this study is unlikely. Furthermore, when only papers with low risk of bias were included in the analysis, the outcomes of this study remained unaffected.

### Literature quality assessment

Additional File 3 shows the quality assessment figure for the 43 studies in this meta-analysis. The potential for bias was broken down into its constituent parts, beginning with the risk of bias associated with the randomization procedure, followed by those associated with deviation from the intended intervention, missing outcomes, the way the outcome was measured, selective reporting, and the overall risk of bias. We found over half of the studies have a low risk of bias, while just a few having a high risk of bias.

## Discussion

In this meta-analysis of 43 RCTs to compare the effectiveness of SGLT2i treatment in reducing the risk of ACS, PAOD, and IS in a total of 79,502 patients with type 2 diabetes (48,568 used SGLT2i treatment and 30,936 used placebo or oral hypoglycemic drugs). For each drug, we covered large RCTs, such as CANVAS (NCT01032629, 4330 people) [20], CANVAS-R (NCT01989754, 5812 people), CREDENCE (NCT02065791, 4401 people) [23] for canagliflozin; DECLARE-TIMI58 (NCT01730534, 17,160 people) [19] for dapagliflozin; EMPA-REG (NCT01131676, 7088 people) [18] for empagliflozin; and MK-8835-004 (NCT01986881, 8246 people) [22] for ertugliflozin. There was no significant difference in the risk of these three diseases between the SGLT2i group and the control group. We examined cardiovascular mortality and all-cause mortality and found that they were lower in the group using SGLT2i, which is consistent with previous studies [24–27], lending support to this study’s validity and indicating that there was no bias in the included trials.

### Implications for SGLT2i in acute coronary syndrome

Our results showed that the use of SGLT2i did not significantly change the incidence of ACS. This is in contrast to a 2017 network meta-analysis by Lee et al. that found SGLT2i to significantly decrease the incidence of ACS compared with the placebo group (N = 6606, RR = 0.50, 95% CI 0.29–0.86)[28], but no significant differences when compared to metformin (N = 1434, RR = 0.66, 95% CI = 0.08–5.64) or sulfonylurea (N = 2264, RR = 0.58, 95% CI 0.17–1.97). Nevertheless, our results vary from those of Lee et al., because four large-scale RCTs conducted after 2017 (N = 35,614) were left out of their analysis.

### Implications for SGLT2i on peripheral arterial occlusive disease

A 2021 meta-analysis by Lin et al. (N = 65,131) found an increased risk of developing peripheral arterial disease (PAD) in patients using SGLT2i hypoglycemic medications (OR = 1.21, 95% CI 1.03–1.42), particularly in patients with canagliflozin (OR = 1.53, 95% CI 1.14–2.05)[29]. Our results showed that the use of SGLT2i did not significantly change the incidence of PAOD in diabetic patients. The key difference between our definition of PAOD and the Lin et al. study’s definition of PAD is that the latter includes 17 specific terms to better explain amputation and diabetic foot-related PAD. In addition, individuals with type 1 diabetes were included in the Lin et al. study, while our emphasis was on those with type 2. Consistent with our findings, another meta-analysis conducted in 2021 by Liao et al. (N = 59,692) indicated that SGLT2i had no effect on PAOD (RR = 1.03, 95% CI 0.75–1.25)[30]. However, sample sizes under 1000 people were not analyzed in Liao et al. study and they included patients other than type 2 diabetes.

### Implications for SGLT2i on ischemic stroke

Zhou et al. conducted a meta-analysis in 2021 (N = 38,723) and showed that the usage of SGLT2i did not substantially change the incidence of IS (RR = 1.04, 95% CI 0.92–1.18) [31], which is in line with our results on IS. Tsai et al.’s meta-analysis (N = 46,969) from 2021 also reported no significant difference for IS (RR = 0.99, 95% CI 0.89–1.12) [32].

There are limitations to this study. Even if heterogeneity is absent (*I*^2^ < 50%), baseline variations in clinical settings, age, follow-up and disease duration may bias the results. We provide both the fixed and random effects model to allow for the clinical heterogeneity seen across several studies. The random effects model produces findings that are comparable to those obtained using the fixed effects model. Second, in order to determine the total occurrences connected to our outcomes, we assumed that studies would use the same definitions of adverse events and inclusion and exclusion criteria. Third, there may be discrepancies in the number of cases reported for the same disease on ClinicalTrials.gov across funding organizations. We may have under-estimated the actual incidence since we applied a strict disease definition to prevent multiple-counting of the same patient. Advantages of our meta-analysis include the fact that it is one of the few to include ACS, PAOD, and IS, as well as the fact that we included both small and unpublished studies found on ClinicalTrials.gov.

## Conclusion

Our meta-analysis of RCTs through November 2022 shows SGLT2i use was associated with a reduction in cardiovascular mortality and all-cause mortality that are consistent with previous research. However, contrary to notions about the cardiovascular effects of SGLT2i, people with diabetes who are treated with these drugs do not have a significantly decreased chance of developing ACS, PAOD, or IS compared to the controls. There is currently not enough data for their meta-analysis to be statistically significant. This may be because of the low incidence of disease in the control group and the modest relative risk reduction for SGLT2i treatment.

## Supplementary Information

**Additional file 1.** Database search algorithm.

**Additional file 2. ** Cochrane risk-of-bias tool (RoB2) used to assess the quality of a study.

**Additional file 3. ** Summary of overall risk of biases in a study.

**Additional file 4. ** Subgroup analysis of studies separated by the presence or absence of atherosclerotic cardiovascular disease at baseline.

**Additional file 5. ** Funnel plots of publication bias for (a) acute coronary syndrome, (b) peripheral arterial occlusive disease, (c) ischemic stroke, (d) cardiovascular mortality, and (e) all-cause mortality.

## Data Availability

The review has not been registered; The dataset(s) supporting the conclusions of this article are included within the article.

## Abbreviations

SGLT2i: Sodium-glucose transporter 2 inhibitors
ACS: Acute coronary syndrome
PAOD: Peripheral arterial occlusive disease
IS: Ischemic stroke
ASCVD: Atherosclerotic cardiovascular diseases
RR: Risk ratio
AE: Adverse events
OR: Odds ratio
HR: Hazard ratio
