# Supplementary material for: Neutral effects of SGLT2 inhibitors in acute coronary syndromes, peripheral arterial occlusive disease, or ischemic stroke: a meta-analysis of randomized controlled trials
Source: Cardiovasc Diabetol. 2023 Mar 13;22:57. doi: 10.1186/s12933-023-01789-5 (PMC10012509; doi:10.1186/s12933-023-01789-5)
Supplement: Supplementary file 1 — Additional file 1. Database search algorithm. [file 12933_2023_1789_MOESM1_ESM.pdf]

Additional File 1. Database search algorithm

("sodium glucose transporter 2 inhibitors"[Pharmacological Action] OR "sodium glucose transporter 2 inhibitors"[MeSH Terms] OR "sodium glucose transporter 2 inhibitors"[All Fields] OR ("sglt2"[All Fields] AND "inhibitors"[All Fields]) OR "sglt2 inhibitors"[All Fields]) AND ("sodium glucose transporter 2 inhibitors"[Pharmacological Action] OR "sodium glucose transporter 2 inhibitors"[MeSH Terms] OR "sodium glucose transporter 2 inhibitors"[All Fields] OR ("sglt2"[All Fields] AND "inhibitors"[All Fields]) OR "sglt2 inhibitors"[All Fields]) AND ("diabetes mellitus, type 2"[MeSH Terms] OR "type 2 diabetes mellitus"[All Fields] OR "type 2 diabetes"[All Fields]) AND ("acute coronary syndrome"[MeSH Terms] OR ("acute"[All Fields] AND "coronary"[All Fields] AND "syndrome"[All Fields]) OR "acute coronary syndrome"[All Fields] OR (("peripheral"[All Fields] OR "peripherally"[All Fields] OR "peripherals"[All Fields] OR " peripheral "[All Fields] OR "peripheric"[All Fields] OR " peripherally "[All Fields]) AND ("arterial occlusive diseases"[MeSH Terms] OR ("arterial"[All Fields] AND "occlusive"[All Fields] AND "diseases"[All Fields]) OR "arterial occlusive diseases"[All Fields] OR ("arterial"[All Fields] AND "occlusive"[All Fields] AND "disease"[All Fields]) OR "arterial occlusive disease"[All Fields])) OR ("ischemic stroke"[MeSH Terms] OR ("ischemic"[All Fields] AND "stroke"[All Fields]) OR "ischemic stroke"[All Fields]) OR ("all-cause"[All Fields] AND ("mortality"[MeSH Terms] OR "mortality"[All Fields] OR "mortalities"[All Fields] OR "mortality"[MeSH Subheading])) OR (("cardiovascular system"[MeSH Terms] OR ("cardiovascular"[All Fields] AND "system"[All Fields]) OR "cardiovascular system"[All Fields] OR "cardiovascular"[All Fields] OR "cardiovasculars "[All Fields]) AND ("death"[MeSH Terms] OR "death"[All Fields] OR "deaths "[All Fields])))
