# Supplementary material for: Neutral effects of SGLT2 inhibitors in acute coronary syndromes, peripheral arterial occlusive disease, or ischemic stroke: a meta-analysis of randomized controlled trials
Source: Cardiovasc Diabetol. 2023 Mar 13;22:57. doi: 10.1186/s12933-023-01789-5 (PMC10012509; doi:10.1186/s12933-023-01789-5)
Supplement: Supplementary file 2 — Additional file 2. Cochrane risk-of-bias tool (RoB2) used to assess the quality of a study. [file 12933_2023_1789_MOESM2_ESM.pdf]

Additional file 2. Cochrane risk-of-bias tool (RoB2) used to assess the quality of a study

	A	B	C	D	E	F
NCT01989754, 2017*	+	+	+	+	+	+
Neal et al., 2015	+	+	?	+	+	?
Perkovic et al., 2019	+	+	+	+	+	+
Lavalle-González et al., 2013	+	+	+	+	+	+
Cefalu et al., 2013	+	+	?	+	+	?
Rosenstock et al., 2016	+	+	?	+	+	?
Bode et al., 2015	+	+	+	+	+	+
Lingvay et al., 2019	+	+	+	+	+	+
NCT01106690, 2013	+	?	+	+	?	?
Yale et al., 2014	?	?	+	+	?	-
Wiviott et al., 2019	+	+	+	+	+	+
R. Henry et al., 2012	+	+	?	+	+	?
NCT01137474, 2014	+	+	+	+	+	+
Wilding et al., 2014	+	+	+	+	+	+
Leiter et al., 2014	+	+	+	+	+	+
Cefalu et al., 2015	+	+	+	+	+	+
Strojek et al., 2011*	+	+	+	+	+	+
Bailey et al., 2015	+	+	?	+	+	?
Bailey et al., 2013	+	+	?	+	+	?
Nauck et al., 2011*	+	+	+	+	+	+
Müller-Wieland et al., 2018**	+	+	+	+	+	+
Scott et al., 2018	+	+	+	+	+	+
Fioretto et al., 2018	+	+	+	+	+	+
Yang et al., 2017	+	?	?	+	+	?
Matthaei et al., 2015	+	?	?	+	+	?
Zinman et al., 2015	+	+	+	+	+	+
Roden et al., 2015#	?	+	+	+	+	?
Häring et al., 2013	+	+	+	+	+	+
Hadjadj et al., 2016*	+	+	?	+	+	+
NCT01649297, 2015*	?	?	+	?	-	-
Ridderstråle et al, 2018	+	+	?	+	+	?
Ferrannini et al., 2013	?	+	+	+	+	?
Barnett et al., 2014	+	+	+	+	+	+
Rodbard et al., 2019*	+	+	?	+	+	?
Rosenstock et al., 2014	+	+	?	+	+	?
Rosenstock et al., 2015	+	+	?	+	+	?
Tanizawa et al., 2015***	+	+	+	+	+	+
Cannon et al., 2020*	+	+	+	+	+	+
Hollander et al., 2019*	+	+	?	+	+	?
Pratley et al., 2018**	+	+	?	+	+	?
Rosenstock et al., 2018	+	+	?	+	+	?
Grunberger et al., 2017	+	+	+	+	+	+
Dagogo-Jack et al., 2017	+	+	+	+	+	+

A	Bias arising from the randomization process
B	Bias due to deviations from the intended interventions
C	Bias due to missing outcome data
D	Bias in measurement of the outcome
E	Bias in selection of the reported result
F	Overall risk of bias

+	Low risk of bias
?	Some concerns
-	High risk of bias
