# Supplementary figures and images for: Neutral effects of SGLT2 inhibitors in acute coronary syndromes, peripheral arterial occlusive disease, or ischemic stroke: a meta-analysis of randomized controlled trials
Source: Cardiovasc Diabetol. 2023 Mar 13;22:57. doi: 10.1186/s12933-023-01789-5 (PMC10012509; doi:10.1186/s12933-023-01789-5)

Additional file 3. Summary of overall risk of biases in a study

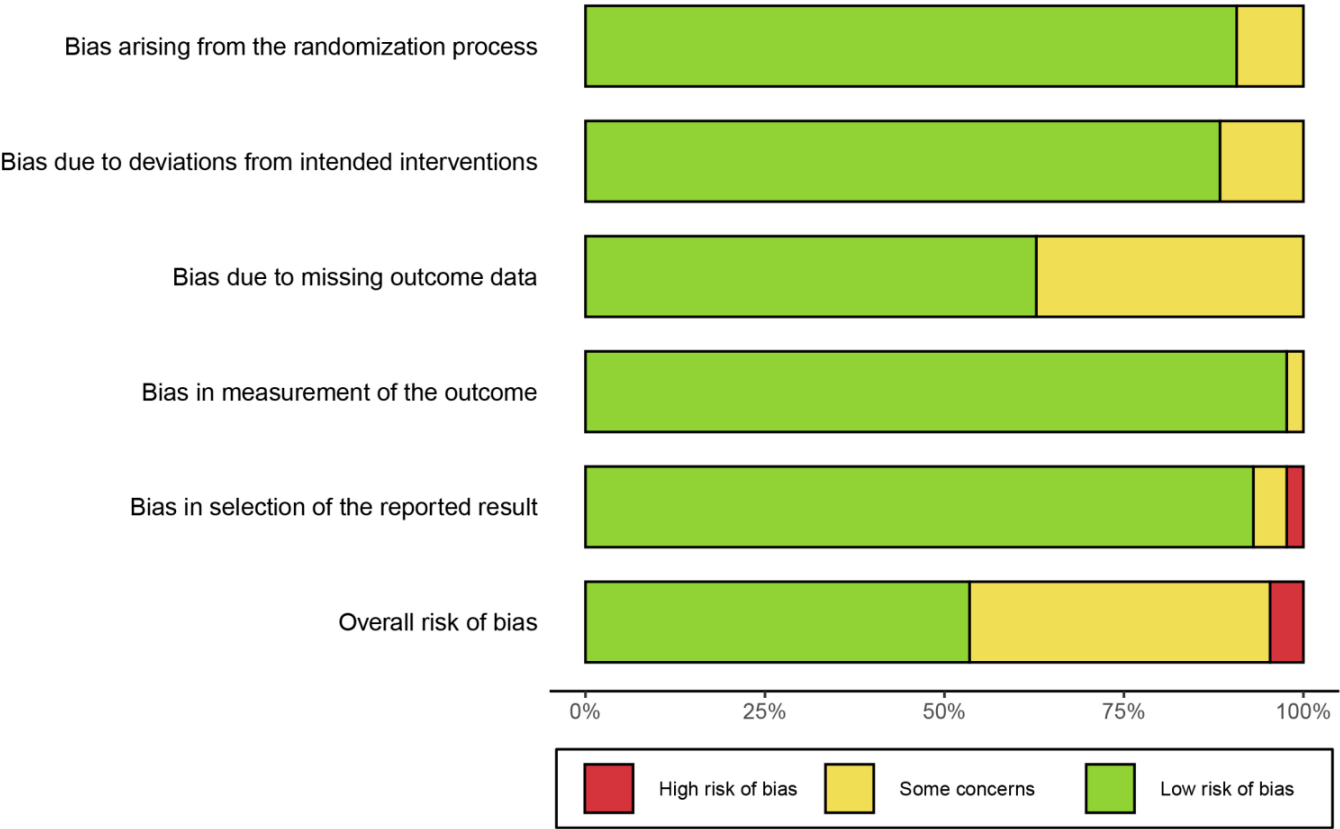

Supplement: Supplementary file 3 — Additional file 3. Summary of overall risk of biases in a study. [file 12933_2023_1789_MOESM3_ESM.pdf]
