# Supplementary material for: Neutral effects of SGLT2 inhibitors in acute coronary syndromes, peripheral arterial occlusive disease, or ischemic stroke: a meta-analysis of randomized controlled trials
Source: Cardiovasc Diabetol. 2023 Mar 13;22:57. doi: 10.1186/s12933-023-01789-5 (PMC10012509; doi:10.1186/s12933-023-01789-5)
Supplement: Supplementary file 4 — Additional file 4. Subgroup analysis of studies separated by the presence or absence of atherosclerotic cardiovascular disease at baseline. [file 12933_2023_1789_MOESM4_ESM.pdf]

Additional file 4. Subgroup analysis of studies separated by the presence or absence of atherosclerotic cardiovascular disease at baseline

	SGLT2i		Control		Risk Ratio* (95% CI)	Heterogeneity I^2	Heterogeneity P-value
	Events	Total	Events	Total			
**Acute Coronary Syndrome**							
Baseline with ASCVD	1116	22100	827	15550	0.97 (0.85, 1.10)	36%	0.18
Baseline w/o ASCVD	184	19781	185	13806	0.87 (0.71, 1.07)	0%	0.93
Overall	1300	41881	1012	29356	0.97 (0.89, 1.05)	0%	0.82
**Ischemic Stroke**							
Baseline with ASCVD	210	22100	158	15550	0.95 (0.78, 1.17)	0%	0.80
Baseline w/o ASCVD	47	14317	42	10573	0.93 (0.62, 1.41)	0%	0.89
Overall	257	36417	200	26123	0.95 (0.79, 1.14)	0%	0.96
**Peripheral Artery Occlusive Diseases**							
Baseline with ASCVD	160	21640	98	15088	1.08 (0.74, 1.57)	46%	0.13
Baseline w/o ASCVD	31	13332	29	9892	0.85 (0.52, 1.37)	0%	0.99
Overall	191	34972	127	24980	0.98 (0.78, 1.24)	0%	0.90
**Cardiovascular Death**							
Baseline with ASCVD	763	19228	571	14120	0.86 (0.65, 1.12)	78%	<0.01
Baseline w/o ASCVD	115	14406	147	9010	0.76 (0.60, 0.96)	0%	0.88
Overall	878	33634	718	23130	0.82 (0.69, 0.97)	28%	0.14
**All-cause Mortality**							
Baseline with ASCVD	1292	20610	1030	14810	0.86 (0.72, 1.02)	66%	0.02
Baseline w/o ASCVD	285	20551	302	13911	0.86 (0.74, 1.01)	0%	0.93
Overall	1577	41161	1332	28721	0.87 (0.81, 0.94)	0%	0.60

*Risk ratios were calculated based on a random effects model.
