# Supplementary material for: Neutral effects of SGLT2 inhibitors in acute coronary syndromes, peripheral arterial occlusive disease, or ischemic stroke: a meta-analysis of randomized controlled trials
Source: Cardiovasc Diabetol. 2023 Mar 13;22:57. doi: 10.1186/s12933-023-01789-5 (PMC10012509; doi:10.1186/s12933-023-01789-5)

Additional file 5. Funnel plots of publication bias for (a) acute coronary syndrome, (b) peripheral arterial occlusive diseases, (c) ischemic stroke, (d) cardiovascular mortality, and (e) all-cause mortality.

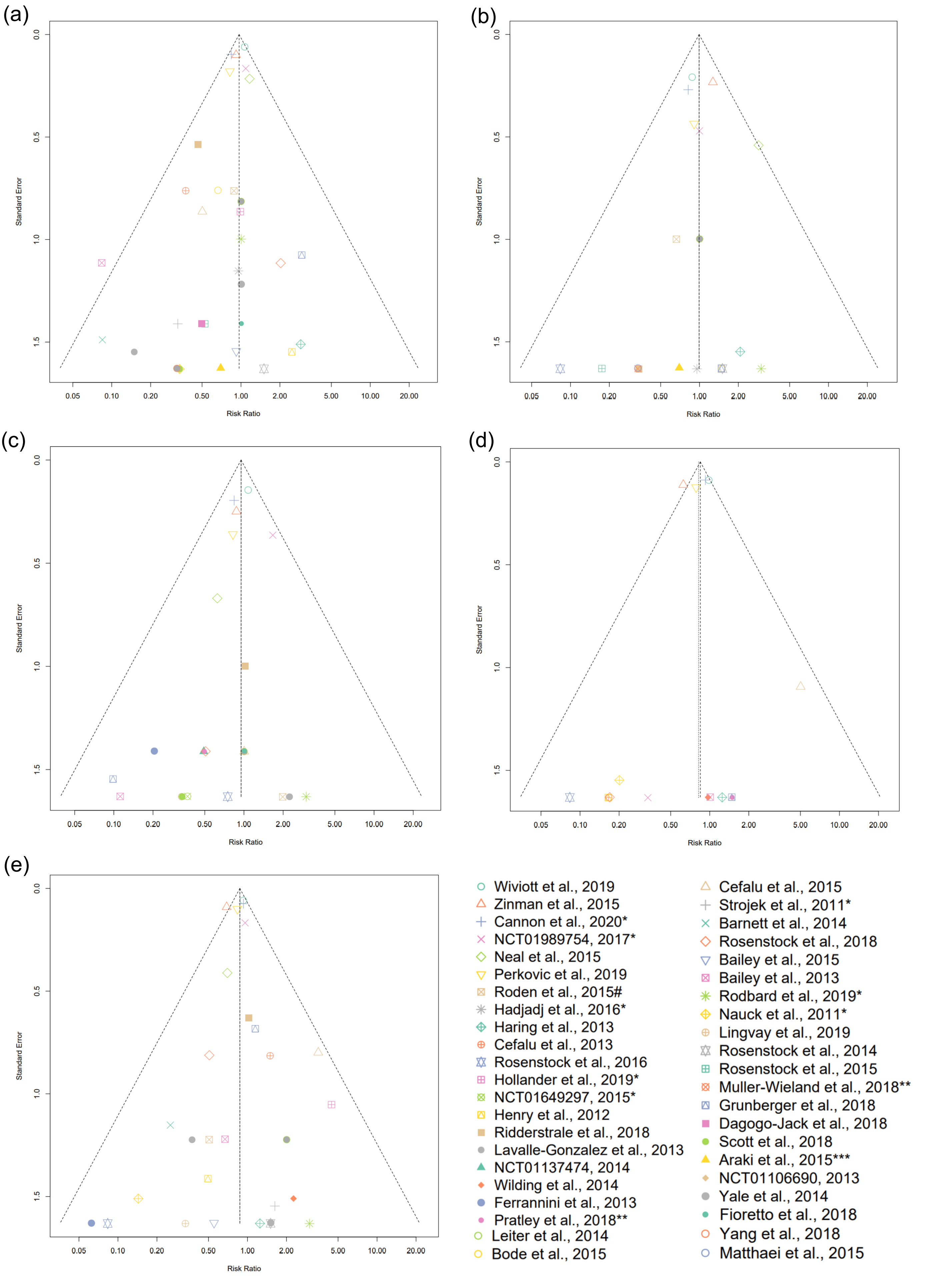

Supplement: Supplementary file 5 — Additional file 5. Funnel plots of publication bias for (a) acute coronary syndrome, (b) peripheral arterial occlusive disease, (c) ischemic stroke, (d) cardiovascular mortality, and (e) all-cause mortality. [file 12933_2023_1789_MOESM5_ESM.pdf]
